# The individual-cell-based cryo-chip for the cryopreservation, manipulation and observation of spatially identifiable cells. I: Methodology

**DOI:** 10.1186/1471-2121-11-54

**Published:** 2010-07-07

**Authors:** Mordechai Deutsch, Elena Afrimzon, Yaniv Namer, Yana Shafran, Maria Sobolev, Naomi Zurgil, Assaf Deutsch, Steffen Howitz, Martin Greuner, Michael Thaele, Heiko Zimmermann, Ina Meiser, Friederike Ehrhart

**Affiliations:** 1 The Biophysical Interdisciplinary Schottenstein Center for the Research and Technology of the Cellome, Bar-Ilan University, Ramat Gan, 52900, Israel; 2 Main Department for Biophysics and Cryotechnology, Fraunhofer IBMT, Ensheimer Straße 48, 66386 St. Ingbert, Germany; 3 WBT Ltd. POB 1516, Ramat Gan, 52115, Israel; 4 GeSiM mbH, Bautzner Landstraße 45, 01454 Großerkmannsdorf, Germany; 5 Zentrum für gynäkologische Endokrinologie und Reproduktionsmedizin, Kaiserstrasse 5-7, 66111 Saarbrücken, Germany; 6 Professorship for Molecular and Cellular Biotechnology/Nanotechnology, University of Saarland, 66041 Saarbrücken, Germany

## Abstract

**Background:**

Cryopreservation is the only widely applicable method of storing vital cells for nearly unlimited periods of time. Successful cryopreservation is essential for reproductive medicine, stem cell research, cord blood storage and related biomedical areas. The methods currently used to retrieve a specific cell or a group of individual cells with specific biological properties after cryopreservation are quite complicated and inefficient.

**Results:**

The present study suggests a new approach in cryopreservation, utilizing the Individual Cell-based Cryo-Chip (i3C). The i3C is made of materials having appropriate durability for cryopreservation conditions. The core of this approach is an array of picowells, each picowell designed to maintain an individual cell during the severe conditions of the freezing - thawing cycle and accompanying treatments. More than 97% of cells were found to retain their position in the picowells throughout the entire freezing - thawing cycle and medium exchange. Thus the comparison between pre-freezing and post-thawing data can be achieved at an individual cell resolution. The intactness of cells undergoing slow freezing and thawing, while residing in the i3C, was found to be similar to that obtained with micro-vials. However, in a fast freezing protocol, the i3C was found to be far superior.

**Conclusions:**

The results of the present study offer new opportunities for cryopreservation. Using the present methodology, the cryopreservation of individual identifiable cells, and their observation and retrieval, at an individual cell resolution become possible for the first time. This approach facilitates the correlation between cell characteristics before and after the freezing - thawing cycle. Thus, it is expected to significantly enhance current cryopreservation procedures for successful regenerative and reproductive medicine.

## Background

With an increasing interest in regenerative medicine, the preservation of living cells has grown in importance throughout biomedical science and the pharmaceutical industry. Cryopreservation is at the moment the only method to preserve living animal/human cells over a long time and it is a method widely applicable in diverse biomedical disciplines, such as reproductive medicine, stem cell research, peripheral and umbilical cord blood cryobanks [1], in the conservation of genetic material from different species [2]. In all these experimental and medical areas, the retrieval of a specific cell or a group of individual cells having particular biological properties is the ultimate objective of the cryopreservation process. Successful cryopreservation of such specific cells is essential for their use in cell-based therapy and research.

There is currently a variety of well-documented cryopreservation protocols [3-7] which have been developed since the discovery of the cryoprotective effect of glycerol [8], dimethyl sulfoxide [9], and 1,2-propandiol which is now frequently used for freezing human gametes [10]. Even though most of these protocols are routinely used, they are incomplete with regard to the survival, functionality, and loss of the thawed cells. These drawbacks might be crucial when the cells are rare (e.g., bio-manipulated cells), and/or highly valuable (umbilical cord blood stem cells, etc.). New experimental techniques and approaches have also been recently developed [11].

As a rule of thumb, the less media exchange the cells experience (while transferring cells between vials containing different media in the process of cryopreservation), the less cell loss there is [12,13]. Nevertheless, in cases when the identity of a cell must be secured throughout the freezing - thawing cycle [14], cryopreservation at a resolution of a single cell must be performed, wherein each cell is stored in a specific macro-vial. In the latter case, the procedure becomes time-consuming, as it takes time to locate the cell in its macro-vial, seize it and transfer it to the next vial. In the cryopreservation of pluripotent and embryonic stem cells, the ability to retrieve a specific colony is crucial both for research and clinical applications, since the characteristics of the cells change during culturing [15] and it is essential to preserve undifferentiated cells [16].

Obviously, if medium exchange can be performed without dislodging/transferring the cells, keeping them in the same primer vial, and if the cells' identity can be secured during these manipulations and throughout the freezing - thawing cycle, e.g. by arresting them, but without interference with their retrieval, then most of the drawbacks of cryopreservation could be overcome. Furthermore, keeping the cells in the same container but spatially separated enables them to maintain chemical cell-cell communication, hence there are no negative effects of cell micro-cultivation.

The present study proposes a new methodology for cryopreservation, using a single macro-vial, in which the medium can be exchanged while maintaining the identity of non-tethered cells before, during and after the freezing - thawing cycle. The proposed methodology is realized via the Individual Cell Cryo-Chip (iCCC/i3C), which was developed in the course of the present study. The i3C enables optical online monitoring, as well as the tracking and retrieving of single cells and colonies. In the present study, the i3C methodology was applied to human pro-monocytic U937 cells only. However, our intention is to implement an adjusted device for the cryopreservation of oocytes and embryos. It will be based on the same methodology but will be adjusted to accommodate large cells.

## Results

### The Individual Cell Cryo-Chip (iCCC/i3C)

**Figure 1:**
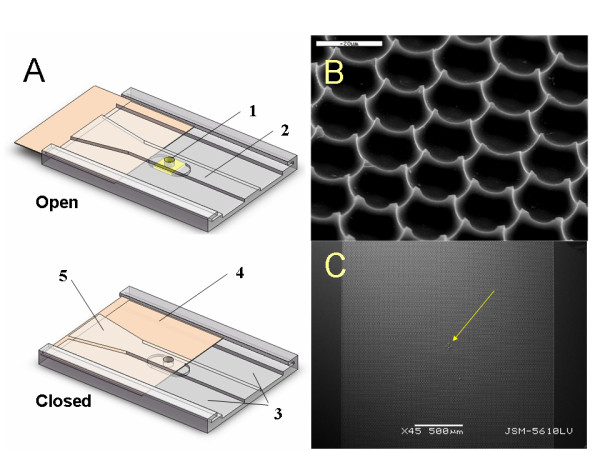
**The i3C and its main components: (1) - cylindrical sample chamber with a bottom made of glass picowells substrate (B and C - SEM micrographs), (2) - elevated flow conduit and its front basin, (3) - two engraved longitudinal grooves, (4) - the sliding cover slip (orange), (5) - waste accumulation area**. Note the reference mark pointed to by the yellow arrow in panel C.

The i3C concept relies on our previous work [17]. However, the i3C was designed and constructed to accommodate the requirements of cryopreservation, namely the freezing and thawing conditions, and to adjust to cryopreservation equipment generally and to cryo-microscopy in particular. The i3C (Figure 1) is a "lab on a chip" capable of retaining cell identity during bio-manipulations and cryopreservation, under continuous microscopic observation. The core of the device is a densely packed 2-D array of Pico-liter Wells (picowells), etched onto a 0.5 mm thick glass in a honeycomb pattern (Figure 1). The latter is a part of a capillary microfluidics system. Each picowell is designed to accommodate a single cell, thus it is shaped with the dimensions of 20 μm opening diameter, 8 μm depth and 20 μm pitch. The picowells' internal surface is smooth, preventing light scattering; its bottom is nearly flat, minimally diverting the wave front of the transmitted light, thus making it optically inert for any practical purposes. In other words, objects located in the picowells can be viewed via a variety of light (upright or inverted) microscopy approaches, including transmitted light (bright field, also enabling tracing unstained cells) and fluorescent light, Differential Interference Contrast (DIC), confocal microscopy, two/multi Photon Exaltation (2PE), etc.

The edges of picowells' walls are sharp (< 0.5 μm in their top/edge), augmenting the surface hydrophilic property and causing the precipitating cells to settle inside the wells rather than in between. The 6 sides of the hexagonal picowell are semi-lunar to allow for the flow of solutions between picowells, while confining each cell within its particular picowell.

**Figure 2:**
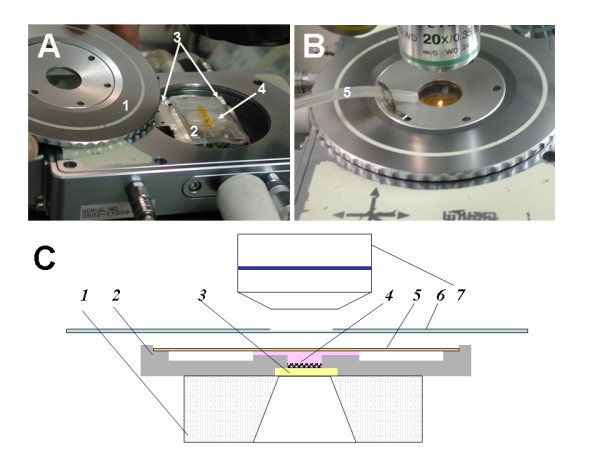
**A) Open cryostage, the lid (1) is detached, the i3C (2, cloudy structure) is attached via its adaptor (3, metal frame around the i3C) to the cooling element (4, circular silver body)**. (B): The lid is closed. Dry nitrogen gas pipe is in place (5). The sample in the i3C is ready to undergo a freezing - thawing cycle. Centers of the lid's window (through which images are acquired - light coming from the condenser is seen) and the i3C's bottom are overlapping and situated on the microscope optical axis. (C) Cross section of the i3C placed on the cryo-stage silver cooling element (1) ready for experiment. The i3C polycarbonate body (2) hosts the glass picowells chip (3) which is secured to its place by bonding. Bottoms are aligned for a perfect contact with the flat cryo-stage. Cells and media (4) which were previously loaded into the i3C are trapped in the cell chamber and the flow conduit due to the capillary action of the sliding cover slip (5). The cryo-stage lid is then closed so that the center of its window (6) and microscope objective (7) optical axis are overlapping to facilitate microscopic observation of cells in the i3C. Note that for the sake of clarity the colors of the i3C components here match those appearing in Figure 1A.

**Figure 3:**
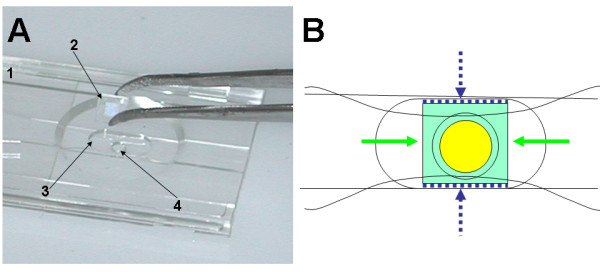
**Attachment of the glass picowells substrate to the i3C base**. (A) The base (1) is placed upside down, and the picowells substrate (2) is gently placed in its socket (3, niche) with its picowells facing down the lower entrance of the cell chamber (4). Next (B): Medical grade UV adhesive (dashed blue) is gently injected in the empty space created between the chip edge and the niche wall, then irradiated and cured. Then, a small drop of liquid PDMS (green) is gently poured near the free edges, and is drawn by capillary forces to fill the empty space between the peripheral face of the chip and the bottom of the niche. The PDMS does not penetrate into the cell chamber area (yellow) due to lack of capillary forces in this open region. When PDMS is cured, the structure is practically sealed to fluids and withstands freezing - thawing cycles.

The picowells-bearing glass chip is embedded under a 0.7 mm deep, φ2 mm cylindrical aperture drilled into an engraved polycarbonate plate, to form the cell chamber. The i3C includes about 7800 picowells of 20 μm. The i3C dimensions (28 × 30 × 2.2 mm^3^) were adjusted to fit the internal dimensions of the cryogenic microscope stage used in this study, enabling the glass substrate to attach directly and fully to the cryo-stage's cooling element (the silver block shown in Figure 2A). The assembly of the picowell substrate to the i3C body is demonstrated in Figure 3.

Materials were selected to withstand the freezing cycles demonstrated here: the i3C body is made of mold-injected polycarbonate with a glass transition temperature of Tg ~ -135°C; the glass substrate is made of high quality BK7 glass. The method of glass to plastic bonding was selected to provide the necessary flexibility required to bridge the difference in their thermal expansion coefficients (polycarbonate: ~70 μm/°C, glass: ~7 × 10^-6^/°C). Partially flexible medical-grade UV adhesive was used to attach the chip to the bottom of the polycarbonate base, to form the cell chamber (loctite 3321, service temperature -55°C). This adhesive is applied only to set the bonding, but does not perfectly seal the connection. The final seal is done with PDMS (GE silicones RTV 655, service temperature ~-115°C) as follows: liquid PDMS is poured around the glass substrate, and penetrates any gaps between the substrate and polycarbonate base. After heat curing (+ 80°C for 1 hour), the PDMS is vulcanized and forms a perfect seal.

**Figure 4:**
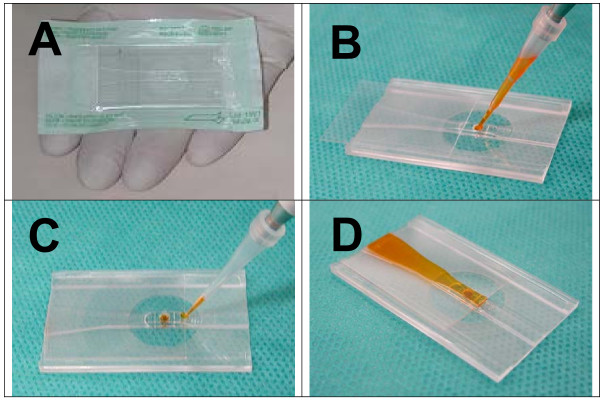
**Working with the i3C: (A) Packed chip in a sterile pouch**. (B) The cover glass is moved back enabling the cell suspension to be loaded into the chamber. The glass is then closed to cover the chamber and to create a short capillary traction area over the input basin (C), so additional aliquots can be added and replace the cell media. Aliquots can be added until the waste area is full and there is no room for additional liquids to enter (D). See text for specific volumes and procedures.

After its assembly, the i3C is packed in a tyvek pouch (Figure 4A) and sterilized in ETO (ethylene oxide) gas.

### Cell loading and administration of solutions into the i3C

The entire procedure is usually performed in a sterilized environment (e.g., sterilized hood, etc.). After removing the i3C from its pouch, its cell chamber is exposed by sliding the cover slip (170 μm thick), and a 7.5 μl suspension of U937 cells in PBS (1.5 - 2 × 10^6^ cells/ml) is poured into it. As the volume of the chamber is about 2.5 μl, a localized spherical drop (of about 5 μl) is formed on top of the cell chamber opening. The cell suspension in the chamber is left for 7 - 10 minutes to allow for cell sedimentation onto the picowell-padded bottom of the chamber. Next, 5 μl of the PBS supernatant (the upper protruding spherical drop) is removed, allowing for the closure of the cover slip, by gently sliding the slip without moisturizing it, leaving a 0.15 mm gap between the cover slip and the conduit.

Next, to insure a complete PBS exchange by the cryopreservation medium (10% Me_2_SO in complete medium), 3 - 4 times the chamber volume (2.5 μl × 3 or 5) is introduced on top of the front basin, against the space between the open conduit and the cover slip. The introduced medium, via capillary forces developed between the open conduit plane and the cover slip above it, travels between the two, along the chamber opening towards the i3C waste area (maximum volume of 30 μl). Through local turbulences developed near the chamber edges, as well as diffusion, the medium reaches the cell layer at the chamber bottom, replacing the remainder of the former buffer.

The end result of this procedure, whether performed in the cryostage or externally, is that cells are suspended in the cryopreservation medium, within the i3C, ready for the next step of incubation in pre-cooling conditions, for further observations, and for undergoing the freezing - thawing cycle. The major steps of cell loading into the i3C and medium exchange are shown in Figure 4. These steps are applicable both within and outside of the cryostage.

Finally, regarding the efficiency of cell loading into picowells, their occupancy and cluster formation in the picowells, observations conducted on a total of 6800 picowells, using cell concentrations of 3.5 × 10^6 ^cells/ml, demonstrated the following: (a) Single-cell occupancy rates were estimated to be 93 ± 2.8% of all the cells located in the picowells. (b) The average percentage of the occupied picowells was about 81 ± 4.2% when 7000 cells were loaded. (c) The distributions of cell occupancy throughout the picowells area and cell-to-cell proximities were found to be homogenous. (d) No cell clusters were observed in occupied picowells. (e) No cell clumping was observed when using non-adherent cells. (f) The coefficient of variance (CV) of cell occupancy measured on 17 different zones within the area of picowells in the chip was 5.1%.

### Evaluation of the i3C ability to retain cells during the freezing - thawing cycle

The cell retaining performance of the i3C during the freezing - thawing cycle was evaluated by monitoring the same cells throughout the cycle using an epifluorescence microscope (Eclipse 80i, Nikon, Japan) which was modified to include a cryostage (MDBCS 600, Linkam Instruments, UK) instead of the regular object table of the microscope. The microscope was equipped with a 100 W Hg fluorescence excitation lamp (LH-m100c-1, Nikon, Japan) and the following fluorescence cubes (excitation filters, dichroic mirrors and emission filters respectively): for fluorescein - 465-495 nm, 505 nm, 515-555 nm, for Hoechst 33342 - 340-380 nm, 400 nm, 435-485 nm, and for PI - 540-580 nm, 595 nm, 600-660 nm.

The silver block of the cryostage (Figure 2) was cooled with liquid nitrogen and heated with an electric heating wire. Temperature was controlled by a thermo-sensor. The control of the heating and cooling rate was performed by a TP94 controller which is connected to a PC. Liquid nitrogen was provided by an external Dewar flask and a liquid nitrogen pump LNP2. For microscopic observation, special objectives are necessary due to the high working distance. Magnifications of 4×, 10×, 20× and 50× were used, where SLWD-objectives provided 20× and 50× magnifications. The i3C was designed to ensure the attachment of the picowells substrate to the silver body, while enabling the conveying, via external adjustment screws, of the field of picowells to the observation area (Figure 2). Nevertheless, the i3C and the cells in it have no direct contact with liquid nitrogen. The only component in the cryostage that is cooled by direct contact with liquid nitrogen is the silver block. This is done internally, via pipes that are carved into the silver cooler, through which the liquid nitrogen is pumped via an external source.

For convenient cell tracing, U937 cells were first (before loading into the i3C) vitally stained with the dyes Hoechst 33342 and QD which label the cell nucleus and the cytoplasm, respectively. A single experiment yielded at least 5 couples of transmitted and fluorescence light images, all of the same field of picowells (20 × 20 picowells), yet each image couple was acquired at a different stage of the experiment: (a) just after cell loading, (b) after closing the microscope lid and pre-cooling, (c) just after thawing, (d) after opening the cryostage lid, in preparation for medium exchange and (e) after post-thawing medium exchange. Dislodged cells were counted by comparing the different groups of images at the individual cell resolution. Images were acquired by the 14-bit ORCA II C4742-98 camera (Hamamatsu, Japan) or PL-A662 - 10 bit RGB color CCD camera (PixeLink, Canada) and analyzed by the cell^R imaging software (Olympus, Japan).

### Sample freezing

After 10 minutes of the stage pre-cooling to 4°C, the first image was acquired and the freezing procedure initiated. A fast cooling rate of 40°C per minute was chosen for one set of experiments and a slow cooling rate of 1°C per minute for another. The freezing end point was -80°C.

Thawing was performed as fast as possible [18]. Generally, thawing continues for less than 3 minutes for full liquefaction. Within the cryostage, the thawing of samples stored either in the i3C or in cryo-mini-vials was always performed controllably: the heating rates were set between 40°C and 30°C per minute. The thawing of samples that were frozen in a standard NALGENE Cryo 1°C Freezing Container was conducted by placing the frozen sample in an incubator at 37°C for 3 minutes. On the average, the latter procedure quite reliably imitates the thawing conditions within the cryo-stage, as the temperature of the frozen sample is elevated from -80° to + 37° in 3 minutes.

Three types of control experiments for the freezing - thawing cycle were performed, each under a different setup. The use of the cryo-stage for either fast or slow freezing control measurements was limited to cryo-mini-vials due to space restrictions. Nevertheless, slow freezing could also be performed within a macro-vial in a cryobox.

In order to achieve the best results with the common cryopreservation, while comparing them with the i3C approach, the ideal conditions for the former were chosen. Therefore, cell suspension without Me_2_SO was not considered as a control, and in all control experiments the cell pellet was suspended in the cryopreservation medium (culture medium and Me_2_SO), incubated for 10 minutes at 4°C, transferred to the freezing device and then subjected to the freezing - thawing cycle. After thawing, the cell suspension was transferred to a 15 ml standard conic tube, and 1-10 ml of fresh complete medium with 20% FCS was added. The cells were immediately washed by centrifugation, and cell viability was optically examined via the i3C:

a) Cells were frozen according to the slow freezing procedure in standard cryo-vials (1 ml cell suspension in the cryopreservation medium) as well as in a non-transparent HDPE cryo-mini-well described earlier [19] (Micro-Cryo well plates, 30 × 25 μl and 9 × 250μl, Fraunhofer IBMT, Germany, 20 μl of cell suspension in the cryopreservation medium). In both cases, freezing was conducted in a standard NALGENE Cryo 1°C Freezing Container (Thermo Fisher Scientific Inc., Waldham, MA, USA) at the rate of about 1°C per minute until reaching -80°C in the Revco Refrigerator (Legaci Refrigeration System, Ashville, NC, USA). Thawing was performed over 3 minutes at 37°C by either placing the sample in an incubator or by immersing it in a water bath.

In summary, cells experiencing slow freezing were cryopreserved for:

1. 15 minutes while in cryo-micro well (in cryostage), and while in cryo-vial (in cryobox) and for

2. 4 days while in cryo-micro well and in cryo-vial (all stored in cryobox).

3. Moreover, cells in the i3C were either kept frozen for 15 minutes within the cryostage or for up to 4 days within the cryobox.

Next, whatever permutations of comparison made between these 6 conditions listed in clauses 1-3, no observable difference was found with reference to post-thawing results groups. Data was analyzed utilizing the ANOVA test for small sample groups. b) Cells were frozen according to the fast freezing procedure, while residing in a non-transparent HDPE cryo-mini-well described earlier [19] (Micro-Cryo well plates, 30 × 25 μl and 9 × 250 μl, 20 μl of cell suspension in the cryopreservation medium). The entire process was carried out in the temperature-controlled cryostage. Thawing was performed in the cryostage as described above.

c) As a principal control, cells from the same culture flask were used, omitting the freezing - thawing cycle.

In all the above cases, cell viability was examined under the same optical conditions, within the i3C, as described above.

Finally, it should be emphasized that, when sterile conditions are required, then after cell loading into a sterilized i3C in a sterilized environment, the loaded i3C is placed into a container, where it is maintained hermetically sealed and sterile during freezing and cryopreservation, either in a Revco refrigerator or in an atmosphere of liquid nitrogen. However, in the present study, even though the cell-loaded i3C can be sterile, the freezing that is performed directly in the cryostage of a cryomicroscope (in contrast to Revco or liquid nitrogen dewar) is conducted in a non-sterile environment. Nevertheless, arrangements can be made to ensure sterility in the cryostage. Even though the importance of sterility for long i3C preservation is obvious, we did not find it critically necessary to assess the feasibility of the i3C methodology.

### Cell retrieval

When the i3C cover slip is open, cells can be retrieved from their picowells by a micromanipulator (e.g., Eppendorf, TransferMan NK2 micromanipulators from Eppendorf, NY, USA, see Additional File 1). However, when sliding the glass cover slip to expose the cell chamber opening for cell retrieval, fluids and cells might be uncontrollably evacuated from the chamber due to adhesion forces between the fluid and the cover glass. This problem was overcome in three ways.

First, only the upper layer of the chamber content that touches the cover slip was thawed, by warming the slip (e.g., by gently touching it from above with a finger) and simultaneously pulling it back, separating the slip from the rest of the frozen content in the chamber and above it. Next, while the slip is open, the thawing of the rest of the frozen content continues without dislodging the cells from their original locations, the cells thus being accessible for retrieval.

Second, thawing was allowed to occur with a closed slip. Then, the slip was opened by applying an intense, short impact on the slip edge in parallel to the slip's movement slots. The sudden thrust pushes the slip back, while the chamber content remains undisturbed due to its inertia and consequently the cells were not dislodged. A simple means to accomplish this was by releasing the head trigger of a ball point pen spring.

Third, while the slip covers the chamber, the open space between the slip and the i3C's surface was flooded with PBS. Next, the slip was pushed to expose the chamber opening for cell retrieval. Flow formation and consequent cell dislodging during the slip movement is prevented, as there is no empty space to be filled by fluids.

### Feasibility study

In slow biological freezing or vitrification processes, all materials exposed to the stressful conditions of cryopreservation must withstand these stresses without breakage or leakage. Matter usually expands with increasing temperature and contracts with its decrease. The heterogeneous cooling/heating of a composite material with different expansion factors causes thermo-mechanical stress. This stress can cause material damage or loss of connection. Furthermore, material elasticity usually decreases with a decreasing temperature, making the material more brittle and vulnerable to other mechanical stresses, such as simple touch or manual/automatic transfer procedures. The durability of the PW substrate and of the i3C body in the severe conditions of cryopreservation was tested as follows.

#### Durability of the picowell glass substrate

Ten glass picowell substrates were challenged in an intense temperature shock cycling test: First, while carried in a metal receptacle, they were immersed in hot water for 3 minutes. Next, after shaking off the water, they were immersed in liquid nitrogen for 3 minutes. After 10 such cycles, the picowell substrates were carefully examined via an atomic force microscope and scanning electron microscope. The substrate structure was found to be intact, including its sharp edges (< 0.1 μm thick) and tips, and its smooth surface. No breaks or small crevices could be observed.

#### Durability of the i3C in the freezing-thawing cycles

Unlike the monolithic glass chip, the complete i3C is constructed of several materials, which have different thermo-mechanical characteristics. Indeed, the i3C design included thermal expansion clearances between adjacent components. However, the durability tests were designed to best reflect the intended use in our experiments. The endurance tests included three repetitions of freezing, thawing and incubating cycles: first, the i3C was loaded at room temperature with a total volume of 12.5 μl of concentrated BO21 in PBS solution (BO21 is a very strong dye that can be easily observed). It was then placed in a -80°C freezer for 1 hour, without any pre-cooling. Next, the i3C was placed over white filter paper to test for leakage. In the leakage test, the i3C was allowed to dry and the paper was inspected for staining marks resulting from leaks. Finally, the i3C, loaded with BO21 and placed in a closed Petri dish, was incubated overnight at 37°C in full humidity. The leakage test was performed again as described. All the tests showed no observable leakage. The i3C thus proved reliable during all the experiments, including freezing down to -80°C and thawing to room temperature.

Complementary i3C-durability tests were performed under extreme conditions as well (not shown here). Briefly, a procedure similar to that described above was used except that the BO21-filled i3C was placed in a small metal box with an embedded thermo-couple sensor and placed into liquid nitrogen. Similar to the experiment with cells, no direct contact was made between the i3C and liquid nitrogen. It took just a few seconds for the measured temperature in the box to reach its steady-state: -153°C. Then, after 10 minutes in liquid nitrogen, the metal box was taken out of the liquid nitrogen, and the frozen i3C was removed from the box. The latter was placed on a clean dry white lab paper, in ambient room temperature for 10 minutes. During this period, the dye in the i3C was fully thawed. Then, the i3C was checked for intactness and leakage as described above.

These freezing - thawing test cycles were repeated 7 times on 3 separate, identical i3Cs. The test results indicate that all three tested samples remained intact, and no signs of leakage were detected.

#### Cell maintenance during the freezing-thawing cycle

**Figure 5:**
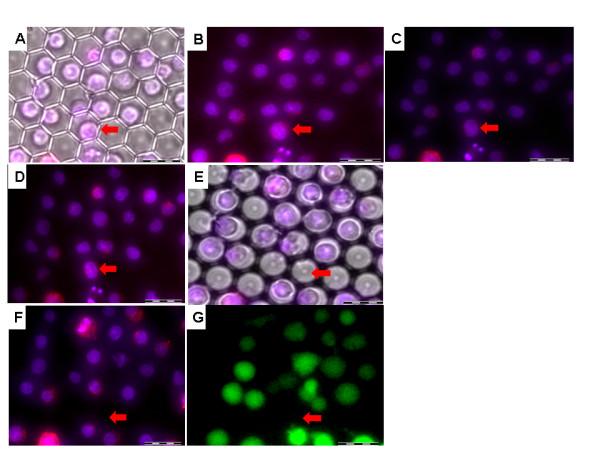
**Monitoring cell retention during the cell freezing - thawing cycle**. U937 cells, labeled with the Hoechst dye (blue fluorescence) and Quantum Dots (red fluorescence), were loaded into the i3C and then underwent a freezing and thawing cycle, which included the following documentation steps: first, before closing the cryostage lid; (A) transmitted light image is superimposed with the fluorescence image of the same field (B). Next, images were taken after closing the cryostage lid (C) and following the pre-cooling step (D), after which freezing was performed. Immediately after thawing, while the lid was still closed, transmitted light (E) and fluorescence (F) images were acquired. To assess the vitality of the same thawed cells, we examined their ability to hydrolyze FDA by exchanging their cryo-media with FDA staining media: 7.5 μm of FDA staining solution was introduced to the conduit basin of the i3C (when the i3C was in the cryostage chamber), and then (after a few minutes) the fluorescence of the FDA-stained cells was imaged (G). Note that the majority of cells retain their spatial location. The red arrow indicates a single cell that changed location after thawing. Bars: 25 μm.

**Figure 6:**
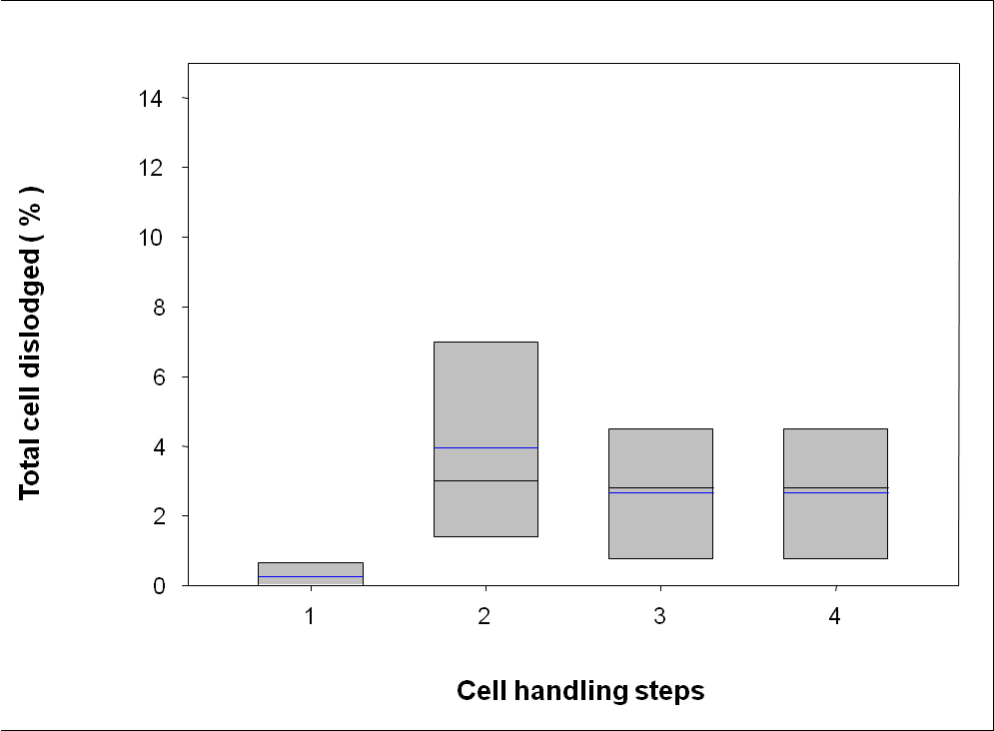
**Cell retention. Box plot of the percentage of dislodging cells during the freezing - thawing cycle**. U937 cells were loaded into the i3C (n = 5), and the same area (20 × 20 picowells) was imaged in the course of cell handling. Total cells dislodging was calculated after each step: 1 - after pre-cooling, 2 - immediately after thawing, 3 - post-thawing after opening the cryostage lid, 4 - after post-thawing medium exchange. The mean value for each data set is indicated by the central line and the first and third quartiles are the edges of the box area.

Under the controlled experimental conditions described above, the majority of cells was found to be maintained in their picowells throughout the freezing - thawing cycle and associated manipulations. A detailed description of a representative cell monitoring experiment is given in Figure 5. The figure includes 7 images of the same field of picowells in the i3C, acquired during the freezing - thawing cycle. The blue and red fluorescences mark the cell nucleus and cytoplasm respectively. This facilitates the identification of the same individual cells when comparing different images obtained in the experiment. In the present study, a total of 5 such comprehensive experiments were performed. They indicate that more than 97% of cells were retained in their original picowells (dislodging < 3%), during the entire freezing - thawing cycle and medium exchange process (Figure 6). Comparing cell locations before the freezing - thawing cycle (step 1) to those that follow it (steps 2-4) showed a significant difference (P < 0.04). However, no statistically significant difference (P = 0.7-0.8) in cell locations was found when all post-freezing - thawing cycle steps were compared. These findings clearly indicated that the main cause for cell dislodging is closely related to the freezing - thawing mechanism.

A similar dislodging percentage was found when the cell-loaded i3C was stored in Revco or in a nitrogen storage tank. Moreover, although the cells may dislodge during the freezing - thawing cycle, whether performed in the cryostage, Revco or nitrogen storage tank (the latter concerns a few cases carried out in this study), they do not dislodge during the further post thawing steps, since the hosting media after thawing is completely converted to a single phase, liquid medium (data not shown).

Apart from these findings, several important practical conclusions were reached regarding the factors influencing cell dislodging (with reference to U937 cells). Generally, the more properly the cells settle (at the base of a picowell), the more spatially stable they are and, consequently, the better they accommodate the forces they are subjected to during the freezing - thawing cycle and medium exchange. Briefly, the factors affecting cell dislodging are the following:

- Suspending medium. The loading of cells into picowells when the cells are suspended in PBS rather than in the cryopreservation medium favors complete cell sedimentation and decreases their dislodging, most likely due to surface tension forces [20,21].

- Loading medium. Cells must be loaded into picowells while suspended in PBS. The loading of cells that had previously been suspended in the cryopreservation medium always resulted in an increased percentage of cell dislodging, presumably due to the higher density (relative to that of PBS) of the medium.

- Settling time. Just after ~10 minutes of cell sedimentation, the initial suspending PBS medium can be exchanged by the cryopreservation medium.

- Cell concentration. This should be adjusted so that a picowell would be occupied by a single cell only. Normally, the first cell to enter a picowell holds the base, forcing the next cell to enter the picowell at a higher position, from which it can be dislodged during the freezing - thawing cycle and medium exchange.

- Heating by illumination. The absorption of transmitted light by the cells and their surroundings is a cause of cell dislodging due to heat agitation. This phenomenon can be considerably reduced by filtering out far-red and near-infrared light. Generally and expectedly, the excitation light used for fluorescence measurements was not found to affect cell dislodging.

#### Cell viability following the freezing-thawing cycle

**Figure 7:**
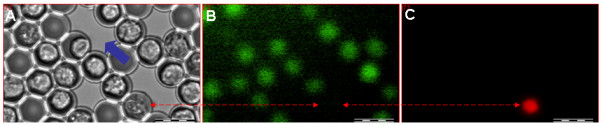
**Monitoring cell viability after the freezing (slow cooling rate, 1°C per minute) and thawing cycle**. Representative transmitted light (A) and corresponding fluorescent images of FDA (B) and PI (C) staining of U937 cells within the i3C after a freezing and thawing cycle. The red line connects images of the only cell in the field found to be PI-positive. The blue arrow indicates a reference region in the picowell field at the bottom of the i3C chamber. Bars: 25 μm.

Under the present controlled experimental conditions, the majority of cells remained alive in their picowells after the freezing - thawing cycle and associated manipulations. Figure 7 shows three representative transmitted and fluorescence light images (of FDA and of PI stained cells) in the same field of picowells. All the images were acquired after a freezing - thawing cycle, the opening of the cryostage lid, and exchange of the cryopreservation medium with a mixture of FDA and PI for vitality assessment, according to the procedure described above. As the figure demonstrates, most of the cells retained their membrane integrity.

**Figure 8:**
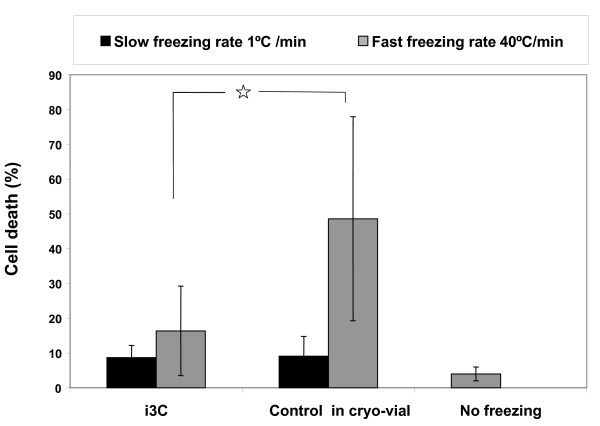
**Cell viability after freezing and thawing within the i3C and in control standard cryo-vials**. U937 cell populations underwent slow or fast freezing followed by thawing in either the i3C or cryo-vials, after which they were exposed, while residing in the i3C or the vial, to staining mixture of FDA and PI. The percentages of dead U937 cells (PI positive) are shown. Bars represent mean and SD values for at least 6 experiments. p < 0.05.

Altogether, 24 experiments were performed to examine cell viability after slow and fast freezing in the freezing - thawing cycle, for cells maintained in the i3C and in a cryo-vial. Samples that did not undergo a freezing - thawing cycle were used as controls. The results are shown in a histogram in Figure 8. The results indicate that, after slow freezing and thawing, the viability of U937 cells within the i3C was the same as in control cells which underwent freezing in standard cryo-vials (the percentages of dead cells were 8.66% ± 3.5 in the i3C and 9.1% ± 5.6 in standard vials). With reference to controls, the comparison between the cryo-mini-vials (in the cryostage) and the (standard) cryo-macro-vials (in the cryobox) showed no detectable differences.

After fast freezing, the variation in cell viability was high. However, while most of the cells did not survive this protocol within the 25 μl micro-vials, a significantly high proportion of cell viability was evident when U937 cells were frozen under the same fast cooling protocol within the i3C (the percentages of dead cells were 16.4% ± 12.8 in the i3C and 48.6% ± 29.9 in micro-vials, p < 0.01). Interestingly, in a limited number of cases, where cell-loaded i3Cs were stored in a nitrogen storage tank, no observable differences were found between the three freezing procedures: cryostage, Revco refrigerator and nitrogen storage tank, regarding the maintenance of cells in the picowells and their membrane integrity.

## Discussion

The present study uniquely demonstrates a new, highly promising approach in cryopreservation, especially successful for the cryopreservation of untethered cells that do not undergo additional treatments in order to avoid cell dislodging. The ability to maintain individual untethered cells in their original positions during the freezing/thawing/post-thawing procedures is the unique feature of this approach which fundamentally distinguishes it from existing devices [11]. This ability was demonstrated on untethered human promonocytic U937 cells under standard laboratory conditions. The heart of this approach is the use of picowell substrates which, to the best of our knowledge, is the first (and presently the only) means that will enable the preservation of intact cells' identity, at the resolution of individual cells, during the freezing and thawing cycle, including medium exchange. Freezing substrates for single cells had been used before [11,19] but lacked either the optical access or microfluidic device that would enable medium exchange without removing the cells. Live-cell bio-banks utilize heterogeneous cell populations whose functional properties need to be preserved. For example, peripheral blood mononuclear cells must maintain their characteristic immune profile, hepatocytes must preserve their metabolic activity [22], and embryonic stem cells must retain their pluripotency after cryopreservation [23]. Hence, successful cryopreservation relies on the ability to observe and retrieve a specific functioning cell or cell group.

The high optical quality of the i3C, as opposed to cryo-vials, enables the observation of samples in the course of cryopreservation (e.g., cryomicroscopy and cryo-multiphoton microscopy [24]) and accompanying treatments. This capability may open a new chapter in cryopreservation, addressing the need to select thawed cells according to their pre-freezing characteristics and to compare pre- and post-freezing - thawing cycle cellular features. Moreover, the i3C in effect acts as a multipurpose single cryo-vial in which the sample (even a few precious cells, instead of bulk-frozen materials) can be fully handled. This method may involve sample preparation (e.g., medium exchange), freezing, thawing, post-thawing treatment (e.g., washing off cryoprotectants, etc.), post-thawing cell culturing and monitoring with no, or only negligible, cell and time loss involved in finding or transferring specific cells. These capabilities make the i3C an ideal means to establish the optimal cryopreservation protocols for various cell types, by defining the correlation between specific cell functions and the optimal retrieval of specific cells after their freezing and thawing.

Regarding the influence of the immediate cell environment (structure, space around the cell, vial material, etc.) that surrounds the sample in cryopreservation, the i3C appears to be dramatically superior to commercial micro-vials in maintaining the viability of cells undergoing fast cooling. Even though the statistical significance of this finding is high, we cannot yet explain it completely. Generally, the surface to volume ratio and the geometry of the cryo-vessel influence the heat ablation, ice crystallization and the extent of possible super-cooling. However, in this case, the properties of the i3C substrates are advantageous for fast cooling (40°C/min). However, in spite of this advantage, the SD of post-thawing cell-death percentage is quite high. The reason for that is intrinsic. Generally, during the freezing of a water solution, the osmollality of the liquid phase is elevated due to the elevation of solutes' concentration. This includes the objects' concentration (such as cells). Consequently, cells in the liquid phase are actually immersed in a liquid, whose solute concentration increases during freezing. Consequently, intracellular water flows from the cells to the hosting solution. In other words, the cells are dehydrating during freezing. Since the freezing of intracellular water damages the cell, the more water leaves the cell before cell freezing, the higher the probability the cell will be preserved through the freezing - thawing cycle, remain alive and properly functioning [25,26]. When slow freezing is applied, intracellular water may have enough time to leave the cell before it freezes. On the other hand, with fast freezing, ice formation in the hosting media considerably precedes cell dehydration. This results in intracellular water freezing and consequently cell death [27]. Actually, the velocity of ice crystal growth is temperature-dependent. The lower the temperature, the faster the growth rate of ice crystals. This leaves the cells less time to dehydrate [28]. For practical purposes, this may be interpreted as if each nucleation temperature results in a "characteristic cell-death percentage."

Next, considering the SD of cell-death percentage, obviously, the more stable the procedure, the lower the resulting SD. When a slow freezing protocol is concerned, the observed nucleation temperature is quite constant: about -15 to -20°C (when using 5-10% DMSO). Obviously, this results in a quite repeatable chain of events occurring during freezing, thus yielding a relatively small SD with reference to the cell death percentage. This is not the case with fast freezing. In contrast to slow freezing, the nucleation temperature is difficult to predict. It may actually vary between -10°C and -40°C. Having established that each nucleation temperature results in its own cell-death percentage, it is expected that fast freezing protocols will yield a relatively high SD.

The micro-vials are designed for automatic application of Me_2_SO which, if included, drastically increases the post-thawing viability of the islets of Langerhans. The mode of application of the cryoprotectant also affects the survivability, so the slow and careful microfluidic medium exchange may contribute to good viability. These experiments provide a first indication for acceptably successful cryopreservation performance of the i3C substrate in comparison to other cryo-substrates. Further experiments are necessary to define the direct causes for their similarities and differences.

Regarding the choice of controls in this study, since this study concerns small cells and many such cells, the suggested i3C methodology is not comparable with vitrification protocols, including cryoloops. On the one hand, the latter are usually intended for fast freezing cryopreservation of only one or a few cells (mostly large and highly valuable cells) and therefore are appropriate for small volumes and require high heat transfer. On the other hand, the i3C is designed to maintain the identity of each individual cell among hundreds, up to millions of small cells, in the same vessel, during the freezing - thawing cycle. Hence, by and large, slow freezing protocols are more suitable for the freezing - thawing cycle performed in the i3C. For this reason we chose for control probably the most widely-used cryo-vessel for cell culture at the moment, the 1 ml cryo-vial, and not cryoloops. Regarding cell retrieval, we found the third procedure described in the "cell retrieval" section to be the most convenient.

Other versions of the i3C are now under construction and/or testing, including adjustments for cryopreservation of large cells, for example oocytes and cell clusters such as spheroids and embryonic bodies. In practice, experiments with oocytes are performed in a specially designed i3C-substrate for oocytes, termed the i3CO (report in preparation). It is the outcome of diverse adaptations to the present i3C and takes into account the size of oocytes, minimal substrate mass and ease of access to oocytes. The oocyte-adjusted picowell has an average opening diameter and depth of ~260 μm and 175 μm (cf. the 20 μm and 8 μm in the present i3C picowells substrate, correspondingly). The new substrate contains only 16 (4 × 4) adjusted picowells, since normally the sample (from the same patient) does not exceed 10 oocytes. Consequently, the mass of the i3CO substrate is about 35 times smaller than that of the present substrate, the i3C. This fact makes the i3CO a low heat capacity configuration, compatible with fast cooling procedures. Obviously, an array of large picowells, such as those of the i3CO, might be used for cryopreservation of cells that are stored in populations, e.g. stem cells.

Furthermore, experiments are being conducted to include the picowell and capillary system in cryo-vials which are especially designed for modern bio-banking (e.g., the "Icebreaker" [29]). Finally, it should be emphasized that, since the present study is a methodological one, the feasibility of the i3C was shown by the use of U937 cells only. Moreover, their durability during a cryopreservation cycle, while residing in the i3C, was determined only by examining their plasma membrane integrity by PI and FDA double-staining. However, due to the importance and broadness of the issue of cell functionality after thawing in the i3C, a comprehensive study has been performed and will be reported separately. In that study, 5 major cell functionality tests were conducted. Briefly, these included: (a) Annexin V versus PI staining for apoptosis and cell death identification; (b) Mitochondrial membrane potential recovery assessed by TMRM staining; (c) Cytoplasm membrane integrity assessed by fluorescein accumulation; (d) Intracellular metabolism tested by FDA staining kinetics; and (e) Cell proliferation. These measurements were all performed both after short (up to 6 hours) and long (up to 48 hours) durations after thawing. Most of these measurements show results similar to those obtained with standard cryo-vials and mini-vials. The remainder show a better performance.

## Conclusion

The results of the present study offer new opportunities for cryopreservation. Using the present methodology, the cryopreservation of individual identifiable cells, and their observation and retrieval at an individual cell resolution become possible for the first time. This approach facilitates the correlation between cell characteristics before and after the freezing and thawing cycle. Thus, it is expected to significantly enhance current cryopreservation procedures for successful regenerative and reproductive medicine.

## Methods

### Biomaterials and probes

Propidium iodide (PI) and dimethyl sulfoxide (Me_2_SO) were obtained from Sigma-Aldrich (St. Louis, MO, USA). Fluorescein diacetate (FDA) was purchased from Sigma-Riedel-de-Haen (Hanover, Germany). Hoechst 33342 trihydrochloride trihydrate dye was purchased from Invitrogen-Molecular Probes (Carlsbad, CA, USA). InGaP/ZnS Evi Tags Quantum Dots (QD), 25 nm hydrodynamic diameter, excitation/emission spectra < 543/650 nm, were obtained from Evident Technologies Inc. (eBiosciencs Inc, Troy, NY, USA). RPMI-1640 medium, heat-inactivated fetal calf serum, penicillin, streptomycin, glutamine, sodium pyruvate and HEPES (all for complete medium) were obtained from Biological Industries (Kibbutz Beit Haemek, Israel).

### Cell culture

Human pro-monocytic U937 cells (ECACC, UK) were maintained in RPMI-1640 medium supplemented with 10% heat-inactivated fetal calf serum, 100 U/mL penicillin, 100 μg/mL streptomycin, 2% glutamine, 2% sodium pyruvate and 2% HEPES. Cells were maintained in completely humidified air with 5% CO_2 _at 37°C. Before use, exponentially growing cells were obtained, washed and re-suspended in PBS at a concentration of 1.5-2.5 × 10^6 ^cells/ml.

### Durability tests

The durability of picowells after the freezing - thawing cycle was examined via an atomic force microscope (AFM - NanoWizard II AFM, JPK Instruments, Berlin, Germany) and scanning electron microscope (SEM - Inspect F50, FEI Company, Hillsboro, OR, USA).

### Cell staining

For easy tracing of cells in the durability experiments, the nucleus was stained by Hoechst 33342 dye (5 μg/ml) in PBS for 1 hour at RT, in the dark. Hoechst 33342 is a very well known vital dye that does not affect cell viability when proper dye concentration and staining duration are used [30-32]. For cell labeling with QD, U937 cells (1.5-2.5 × 10^6 ^cells/ml) were incubated in the presence of 25 pM QD solution (this concentration was found to be non-toxic), in complete RPMI medium at 37°C for 3-48 hours in a completely humidified atmosphere with 5% CO_2_. At the end of incubation, cells were washed with fresh medium and re-suspended in PBS.

In order to determine plasma membrane integrity for the cell viability assay, cells were double-stained with PI and FDA. A mixture of FDA (final concentration 1.2 μM) and PI (final concentration 2.5 μg/ml) was added to U937 cells loaded into the i3C, for 5 minutes in the dark, at RT (the method is similar to that reported by Ehrhart et al. 2009 [33]). The cells were then imaged using fluorescence cubes for fluorescein and PI.

### Statistical analysis

For each experiment, 2-3 i3C devices were used. For each i3C device, 5 images from 5 different areas were acquired (about 800-1000 individual cells). Each area was imaged 4 times: before freezing, immediately after thawing, after cryo-stage opening and following medium exchange, as described in the section "Evaluation of the i3C ability to retain cells during FTC - the experimental procedure." The mean and SD for each measured parameter were calculated for the different cell populations under investigation (control and experiment). Comparisons between cell groups (controls and experiments) were carried out using the ANOVA test for small sample groups. All the values are represented as average ± SD.

## Competing interests

The authors declare that they have no competing interests.

## Authors' contributions

EA participated in the experimental design, carried out fluorescence measurements during the freezing - thawing cycle and participated in the process of image analysis and drafting of the manuscript. AD led the design and construction of the i3C, participated in the durability tests and SEM examinations, produced sketches of the manuscript and edited its figures. MD conceived the individual cell-based Cytometry approach, drafted the manuscript and, together with HZ, designed and coordinated this study. HZ conceived the individual cell-based cryopreservation approach and, together with MT, identified its advantages for reproductive medicine. FE participated in the experimental design, carried out fluorescence measurements during the freezing - thawing cycle, performed durability tests and AFM examinations, performed the literature survey and helped to draft the manuscript. MG participated in performing the complementary control (routine) freezing - thawing cycle experiments. SH contributed to the microstructure design and manufacturing aspects of the i3C. IM was responsible for the various regimens of freezing - thawing cycles and relevant controlling S/W programs, and contributed to the analysis of images. YN programmed the S/W which automatically identified populated/empty picowells, stained and unstained cells in the picowells, and prepared the acquired data for the 2^nd^/3^rd ^party analysis. YS carried out fluorescence measurements during the freezing - thawing cycle, prepared samples for SEM examinations and participated in the durability tests. MS carried out fluorescence measurements during the freezing - thawing cycle and participated in the image analysis. NZ led the biological and image analysis parts of the study, participated in designing the study and drafting the manuscript. All authors read and approved the final manuscript.

## Supplementary Material

Additional file 1 **Selective cell retrieval using a micro-manipulated glass capillary (see text for details)**. Click here for file
